# Comparative effectiveness of financing models in development assistance for health and the role of results-based funding approaches: a scoping review

**DOI:** 10.1186/s12992-023-00942-9

**Published:** 2023-06-20

**Authors:** Rand Mushasha, Charbel El Bcheraoui

**Affiliations:** 1 Institute of Tropical Medicine and International Health, Charité – Universitätsmedizin Berlin, Augustenburger Pl. 1, 13353 Berlin, Germany; 2 Evidence-Based Public Health, Centre for International Health Protection, Robert Koch Institute, Nordufer 20, 13353 Berlin, Germany

**Keywords:** Development assistance for health, Global health financing, Results based financing

## Abstract

Over the past three decades, there has been an unprecedented growth in development assistance for health through different financing models, ranging from donations to results-based approaches, to improve health in low- and middle-income countries. Since then, the global burden of disease has started to shift. However, it is still not entirely clear what the comparative effect of the different financing models is. To assess the effect of these financing models on various healthcare targets, we systematically reviewed the peer-reviewed and gray literature. We identified 19 studies and found that results-based financing approaches have an overall positive impact on institutional delivery rates and numbers of healthcare facility visits, though this impact varies greatly by context.

Donors might be better served by providing a results-based financing scheme combining demand and supply side health-related schemes. It is essential to include rigorous monitoring and evaluation strategies when designing financing models.

**Supplementary Information:**

The online version contains supplementary material available at 10.1186/s12992-023-00942-9.

## Background

Development assistance for health (DAH) is defined as “financial and in-kind resources that are transferred through major international development agencies to low- and middle-income countries with the primary purpose of maintaining or improving health” [1]. The past two decades have witnessed an extraordinary growth in DAH [2]. The global burden of disease started to shift in the last decades, undoubtedly reflecting the effect of DAH disbursements. DAH is allocated through different financing models such as direct funding (i.e. donations), performance-based financing and results-based aid, among others [3]. The disbursement is usually aimed at various different health areas such as HIV/maternal health/tuberculosis, healthcare quality and vaccinations to name a few. Bilateral, multilateral, and other donors’ disbursements increased from $12 billion in 1999 to $32 billion in 2009. In the ‘peak days’ between 2000 and 2010, the yearly growth rate was more than 11% on average, and DAH grew almost three times faster than development assistance to non-health sectors [4]. However, DAH has slowed down, with annual growth from 2010 to 2018 estimated at 1·33% [6] and only $8 billion additional financing support was provided in 2019 in comparison to previous increases[5, 6]. This drop can be attributed to some countries graduating from multilateral development assistance as they have reached national gross domestic product per capita that does not make them eligible for aid [7]. However, this trend reversed quickly in the following year, with an additional 15$ billion being disbursed within the first year of the COVID-19 pandemic, of which 14$ billion were allocated to COVID-19 [6].

One issue arising with foreign aid is that donors might have goals that are more likely to serve their own interests rather than recipient needs, such as providing health aid to protect their own population by targeting infectious diseases that spread rapidly, such as COVID-19, or promoting their political and economic interests [8, 9].This major drop followed by the massive increase since the beginning of the pandemic stresses the importance of increasing the efficiency in any present and future DAH, as well as identifying the most effective financing models that could optimize health systems’ preparedness and population’s health.

Up till now, DAH-based programs have yielded different levels of health improvement and outcomes. The lack of a results’ based focus is considered by many a major reason why past aid efforts have produced disappointing results [10]. In this context, donors are looking for ways to scale up support while, at the same time, show the results their aid is achieving. Hence the interest in the role that results-based funding might play [11], and therefore our emphasis on result-based approaches in DAH. It is important to note that Results Based Financing (RBF) depends greatly on country context, and at any times can be a mix of domestic and DAH sources. Defining results-based approaches is troublesome. Issues in definition are magnified by the often-misleading terminology used by the various schemes. We generally follow the working definitions adapted from the World Bank’s Global Partnership for Results Based Approaches (GPRBA) and have applied them to official development aid for health. Under the umbrella of Results Based Financing are various financing models implemented in DAH, some are outlined below:

1. Cash on Delivery Aid, which is also considered Results Based Aid (RBA), is a fixed payment to the government for each unit of result delivered. The unit is specified in a contract between the donor and a government.
2. Conditional cash transfers (CCT), which are a typical demand-side RBF tool where a predefined amount is given to targeted populations for complying with certain requirements or accessing a certain health service.
3. Performance based financing (PBF) which is a supply side targeting tool where healthcare providers are incentivized to deliver good performance of different services. The principal and agent often collaborate in establishing performance indicators to evaluate goals accurately. The terms performance-based financing and pay for performance (P4P) are used synonymously in literature. Furthermore, performance-based contracting (PBC), is a specific case of PBF that includes a more detailed contract specifying a fixed price for a certain desired output and is typically applied to non-governmental organizations (NGOs)[12, 13].

Fundamentally, demand side schemes are schemes targeting individuals who utilize health care services. On the other hand, supply side schemes target service providers. Combined schemes aim to support both the supply and demand sides of a specific service[14].

While multiple studies have attempted to quantify the impact of DAH on the burden of disease, as well as the amount of financial support given towards certain health areas and regions, the comparative effectiveness of the different DAH models is rarely addressed, leaving much room for uncertainties among donors on choosing the mechanisms for their financial contributions. We systematically reviewed the available evidence on existing DAH financing models to compare their effectiveness, and to help donors better allocate potential resources available for DAH.

## Methods

To compare the effectiveness of models of financing DAH, we systematically reviewed the literature to identify and analyze peer-reviewed articles that study the effect of such models. We searched scientific peer-review databases and other sources for evidence on the effectiveness of the different financing models of DAH.

### Data Search

Initially, we used the PubMed database to extract relevant literature. We conducted the search on January 19,2022. We carried out a second search using the Embase database on January 30,2022.

The following descriptive terms were used for both searches:

*((((((((((((financing) OR (“financial aid”)) OR (“global health”)) OR (“financial model”)) OR (aid))) OR (“effectiveness”)) OR (performance based aid)) OR (performance based financing)) OR (result based aid))) OR (“development bank”)) OR (“donor”)) AND (“development assistance for health”)*.

Results were filtered to include documents written in the English language in the last 20 years. The search was tailored to include only original research articles or book sections, thereby excluding preprints, editorials, letters to editors, commentaries, interviews, and correspondence. Articles were removed during the title and abstract screening stage if the title did not address the topic of interest. We then retrieved the full text of the potentially relevant articles and conducted a full text analysis of the studies to assess eligibility using predefined inclusion and exclusion criteria below.

### Inclusion criteria:

- Gave a general overview of DAH financing models.
- Studied the effects of a certain financing model (i.e.: RBF, RBA).
- Were case studies of projects implementing one of the above financing models in low income and lower-middle income countries (LMICs).

### Exclusion criteria:et

- They did not specify the financing model(s) of DAH implemented.
- The study setting did not include LMICs according to the World Bank Open Data (https://data.worldbank.org/).
- They did not provide a health-related outcome and were limited to quantifying disbursement of funds.

Citation harvesting was implemented in the selected studies to track citations that could be relevant for our review.

### Identification of documents via other sources

In addition to the peer-reviewed literature, we sought to find information on each of the financing models of interest separately based on the inclusion and exclusion criteria previously specified. Therefore, websites of international development institutions and public health research institutes were explored to find further analyses on DAH using the following key terms: *Development Assistance for health/DAH, result based aid/RBA, and result based financing/RBF*.

The following websites were used in the gray literature search:

- GAVI The vaccine Alliance (https://www.gavi.org/programmes-impact/our-impact/evaluation-studies).
- The Global Fund to Fight AIDS, Tuberculosis and Malaria (GFATM) (https://www.theglobalfund.org/en/publications/).
- United States Agency for International Development (USAID)(https://www.usaid.gov/global-health).
- World Health Organization (WHO)(https://www.who.int/data/gho/publications).
- United Nations Children’s Fund(UNICEF)(https://www.unicef.org).
- Organisation for Economic Co-operation and Development (OECD)(https://www.oecd.org/health/).
- Center for Global Development (CGD)(https://cgdev.org).
- German Development Institute of Development and Sustainability (IDOS)(https://www.idos-research.de/en/).
- Global Partnership for Results-Based Approaches (GPRBA)(https://www.gprba.org/knowledge/resources).
- World Bank, RBF Health (https://www.rbfhealth.org).

This grey literature search provided a number of working, discussion and policy papers covering certain financing models.

### Data extraction

We categorized the studies according to type of financing model and entered data from the selected studies into a form created for that purpose on Microsoft Excel 365® software for Windows in tabular form. The following data were charted: author, publication year, study design, study methods, timeframe, financing model type, recipient, funding target, mode of disbursement, amount of disbursement and funding source.

Data extraction form can be found in additional file 1. EndNote software was used to sort, arrange, and cite the included studies.

## Results

**Fig. 1:**
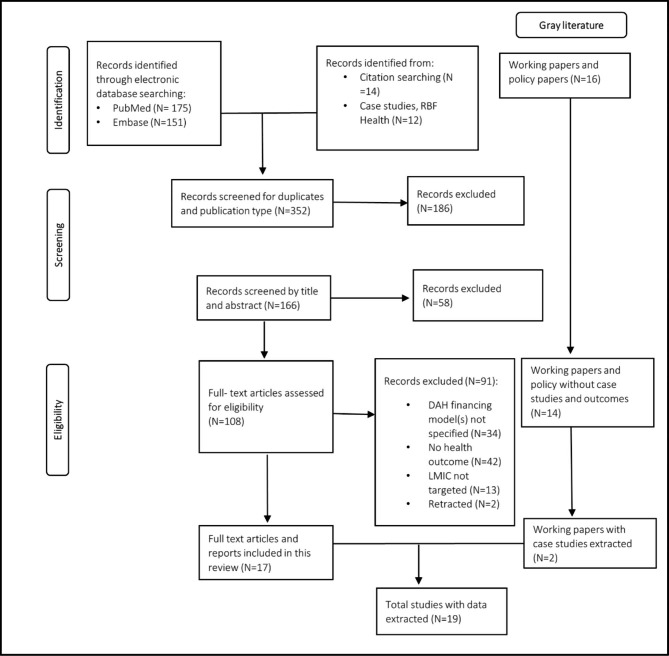
PRISMA flowchart for selection of data

We followed the Preferred Reporting Items for Systematic Reviews and Meta-Analyses (PRISMA) statement shown in Fig. 1 below[15]. In total, we identified 19 studies which we included in this scoping review. Of the 19 studies, 17 were peer-reviewed articles, and two were working papers identified through the gray literature search. Of the 17 peer-reviewed articles, six were systematic reviews, one was a qualitative study, one was a randomized controlled study and nine were quasi-experimental studies. More information on each study design is included in the data extraction form in Additional file 1. Oxman *et al. *[16] reviewed RBF as well as the Immunization Service Support (ISS) program implemented by Gavi, the Vaccine Alliance (GAVI). Eight of the nine quasi-experimental studies targeted RBF. Mokdad *et al.* [17] conducted a quasi-experimental study on the Salud Mesoamérica Initiative (SMI), an initiative with a RBA model. There was one qualitative study assessing PBF.

In this section, we present the outcomes of each of the RBF schemes, aggregated by recipient type. Table 1 shows outcomes of schemes targeting the demand side such as CCTs and non-monetary incentives, Table 2 shows the outcomes of schemes targeting combined demand and supply side schemes such as vouchers and Table 3 shows the outcomes of schemes targeting supply side schemes, which are performance-based financing models. Additional file 2 shows the outcomes aggregated by indicator.

**Table 1:** Outcomes of demand side schemes

Country	Performance indicator(s)	Outcome(s)
CCT		
Malawi (40)	HIV testing rates	Mean percentage increase by 27%, positive linear effect with level of incentive
Nigeria(29)	Achieving four antenatal care (ANC) visits, institutional delivery as well as DPT3 and measles immunization of children	Increased utilization of institutional deliveries and rates of ANC visits but no significant effect on completion of child immunization using measles as a proxy indicator
India(37)	Institutional delivery rates	With-versus without comparison analysis: reduction of 4.1 perinatal deaths/1000 pregnancies and 2.4 neonatal deaths/1000 livebirths
(28)	Behavior improvement and patient motivation	Increased case detection and 100% treatment completion
Nicaragua(40)	Child health indicators	• Net mean improvement of the height- for-age z score by 0.17 after 2 years • Net impact of 6 pp in proportion of underweight children aged 0 to 5 years • No impact on anemia prevalence among infants • Mean increase in proportion of infants aged 0–3 years taken to health centers in the past 6 months
Honduras(40)	Monthly preventive health examinations for children rates	• Increase mean percentage of individuals receiving pre-natal care by 19 pp • Routine pediatric examinations by 20 pp, growth monitoring by visits for children by 16 pp • Significantly increased use of health services by 23% for infants younger than 1 year and 42% for preschool children aged 1 to 5 years
	Prenatal care attendance for pregnant women rates	No effect on percentage of women who received a 10-day follow-up visit after delivery
Non-monetary incentives		
Tajikistan(16)	Tuberculosis treatment adherence and cure rates	• 30.1% difference in cure rates between vulnerable group and control • Treatment failure was 3.9% in the food support group vs. 15.6% in the comparison cohort
Cambodia(28)	Tuberculosis treatment adherence and cure rates	Cure rate = 92%; default rate = 2%
Sudan(28)	TB treatment completion	Treatment completion rate of 82% partially attributed to scheme
Yemen(28)	Clinic attendance for TB treatment	Cure rate for areas with food packages: 85% compared with 78% without

**Table 2:** Outcomes of combined demand and supply side schemes

Country	Performance Indicator(s)	Outcome(s)
PBF&CCT		
Malawi(42)	Maternal mortality rates	Reduced facility based maternal mortality by 4.8 deaths/100 000 facility -based deliveries
India(16)	Rate of institutional deliveries and ASHA promotion of primary healthcare services	• Proportion of institutional deliveries increased from 32.5–65.1% • 43% of ASHA were satisfied and 36% were somewhat satisfied with the remuneration received
Nicaragua(27)	Percentage of children with updated vaccinations and percentage of children under 5 years old with stunting	• Percentage of Children 12–23 months with updated vaccinations Baseline: control: 41.5% intervention: 38.9% Follow up: control: 69.4% intervention: 71.4% • Percentage of stunted children under 5: Baseline: control: 39. 5% intervention: 39. 8% Follow up: control: 41.7% intervention: 36.5%
Uganda(22)	Maternal maternity rates, demand for health facility births	• 9.4% bump in institutional delivery implying 20 deaths averted, which is equivalent to 1356 disability- adjusted-life years (DALYs) averted • Demand for births at HFs enrolled in the voucher scheme increased by 52.3 pp. Out of this value, conservative estimates indicate that at least 9.4 pp are new HF users

**Table 3:** Outcomes of supply side schemes

Country	Performance Indicator(s)	Outcome(s)
PBF/P4P		
Democratic Republic of Congo(20)	Maternal and child health services provided	• Did not improve full immunization among children and anti-tetanus vaccination (VAT2þ) among pregnant women • Improved assisted delivery (42%) • Relative increases in curative care (83%) • Increased HIV/AIDS testing among pregnant women (147%) • Increase in patient referrals (472%) • Vitamin A distribution (155%)
Burkina Faso: North (Titao), Center-North (Boulsa), and Center-West (Leo)(25)	Maternal health services: number of antenatal care (ANC) visits, proportion of ANC visits during first trimester, number of complicated and uncomplicated deliveries at HF and number of postnatal consultations provided 42 days after pregnancy	• Relative increase of 9.2% for deliveries • Relative increase of 27.7% for ANC visits • Relative increase of 118.7% for postnatal care visits
Mozambique: Nampula (North) and Gaza (South)(34, 35)	Provision of ART for pregnant women, prevention of mother-to child HIV transmission (PMTCT), and maternal/child health (MCH), health worker motivation	• Women completing 4 ANC visits: North 153% increase over baseline, South 82.4% increase over baseline • Initiation of ART for pregnant women with HIV: North 251. 6% increase over baseline, South 194.6% increase over baseline • Internal drivers: enhanced self-efficacy driven by goal orientation, healthy competition among colleagues, and job satisfaction. External drivers included an organized work environment, enhanced access to equipment and supplies, financial incentives, teamwork, and regular consultations with verifiers
Haiti(24)	Increase of health services provided	• Incentives alone were associated with a 39% increase in health services • Support alone was associated with a 35%increase in health services • Support and incentives were associated with an 87% increase compared with health facilities that did not receive either
Rwanda(24)	Maternal and childcare services provided, quality of curative, maternal and child health, and HIV/AIDS services	• No improvement in the number of children receiving full immunization schedules • 23% increase in institutional deliveries compared to control • 56% increase in preventive care visits by children aged 23months or younger • 132% increase in preventative care visits by children between 24 and 59 months, compared to the control group • No improvement in the numbers of women receiving any prenatal care, the number of women completing four or more prenatal visits
Rwanda: Cyangugu, Butare(27)	Quality increase of curative, maternal and child health, and HIV/AIDS services	• Measles immunization: 10.8% increase • Institutional deliveries 10.9% increase • Curative care quantity: increase of 0.33 per person per year • Family planning acceptors: 2.8% increase
Rwanda: Kigali- Ngali, Kabgayi, and Kigali Ville(27)		• Measles immunization: 3.6% increase • Assisted deliveries: 8.5% increase • Curative care quantity: 9.7% increase • Family planning acceptors: 5.1% increase
Bangladesh(27)	Tuberculosis case detection and treatment adherence rates	Case detection rates = 50%, cure rates = 89%, partially attributed to incentive scheme
PBC		
Haiti(32)	Maternal and child health, reproductive health, and family planning services	• Full immunization coverage :11.3% increase • Assisted deliveries: 2% increase • Pregnant women receiving at least one prenatal visit: 55.5%
(31)	Immunization coverage rates and uptake of services	• Increased immunization coverage by 32% • Increased oral re-hydration salts usage
Cambodia(31)	Utilization of healthcare services	• Increased utilization by the poor, decreasing total family health expenditure from US$18 to US$11 per capita per year
PBF&PBC		
Cambodia(23)	Rate of births in health facilities	Estimated to raise the probability of births occurring in incentivised public health facilities by 7.5 percentage points

Narrative systematic literature reviews included in our study gave a wide range of results. Beane et *al*’s [13] systematic literature review of PBF models has shown that programs in the Democratic Republic of Congo, Egypt, Burundi have positive and mixed results and a PBF program in Tanzania proved to be less successful. The review’s assessment of a PBC programs showed an effective increase in service delivery in Cambodia. They also stated that a PBC model in Liberia and Afghanistan showed promising results. On the other hand, PBC faced challenges in Sudan and had no effect in Uganda. However, a more recent study published by SSengooba *et al.* shows that Uganda’s health policy agenda has given increased attention to RBF. Plausible explanations included external aid, national ownership, increasing capabilities for implementation and stewardship of RBF programs [18].

To summarize the results of the systematic review by Diaconu et *al*[19]: When not targeted, P4P probably slightly reduces child mortality, the proportions of children with anemia and children with wasting with moderate-certainty evidence. When targeted, the effects of P4P on the delivery and utilization of services was inconsistent: the intervention may improve some services’ delivery and utilization. Indicators such as proportion of people receiving HIV testing, delivery of prevention of mother-to child HIV transmission (PMTCT) and family planning outreach was positive but other indicators such as proportion of people receiving antiretroviral therapy (ART) and proportion of households protected by bed nets showed poorer results. P4P may improve the quality of targeted services and overall, P4P may have desirable effects on resource use when targeted. PBF may improve the quality of targeted services and overall may have desirable effects on resource use when targeted. According to the authors of this paper, most of the evidence is of low certainty and more well conducted studies are needed. Results with very low certainty were excluded.

When looking at aid given to governments, Oxman *et al.* [16] included an evaluation of the first 5 years of GAVI’s Immunization Services Support Funding (ISS) in 2010. It is estimated that GAVI funds increased immunization program funding 15% from pre-GAVI levels. The primary outcome was improvements in immunization coverage. Other outcomes include impact on overall immunization financing, cost per additional child vaccinated, and equity. Overall, the study concluded that a relationship was found between ISS funding and increased immunization coverage. On the other hand, the study by Zeng *et al.* found that RBF did not improve full immunization among children and anti-tetanus vaccination (VAT2þ) among pregnant women in the Democratic Republic of Congo [20].

Mokdad *et al.* [17] provided findings from SMI. SMI was designed to target disparities in maternal and child health, focusing on the poorest 20% of the population across the region. Participating countries receive 50% of the cost of program intervention and contribute the remaining 50% themselves. If 80% of the performance indicators are met, the country is awarded its contribution back. There were multiple health facility indicators incorporated. All countries had progress in the indicators, although with different levels. Countries that did not reach their 18-month target did reach them 9–12 months later. The study concluded that the RBA approach can be a driver to improve availability of drugs and services in poor areas.

## Discussion

Implementing a results-based approach is currently on the forefront of development assistance for health. Results based financing approaches show promise but the literature is still significantly lacking, especially in LMIC settings. It is essential to note that the effectiveness of the different results-based approaches varies greatly by context and health target. Our research provides a unique and original contribution to the existing literature, as we aim to accurately define, categorize and distinguish between results-based financing models. Furthermore, we aim to assess the potential effectiveness of these models in different contexts, as contextual factors significantly shape the design and outcomes of such models. Currently, no comprehensive review on this topic exists, and despite our exhaustive research, further investigation is required to expand our knowledge on this subject matter. In our scoping review of 19 studies of different methodological designs, the most frequent target studied in the literature we have selected was maternal and child health. The studies also indicated that immunization rates and HIV/TB cure rates and treatment completion were of significant interest. Among the impact indicators frequently utilized in the studies, rate of institutional deliveries was the most frequent, followed by immunization rates in children. However, there is a significant funding gap for maternal health globally and this gap underscores the need for increased investments in maternal health to improve health outcomes for both mothers and children [21].

In terms of improving supply side indicators, PBF predominantly showed a positive effect in increasing rates of institutional deliveries [20, 22–28] —the greatest rate of increase was in regions with a lower baseline in maternal health services— but mostly not on children’s immunization rates [20, 29] and in some cases, had a negative effect on immunization rates [20]. This limited impact could be attributed to limited availability of vaccines [20, 27]. According to the recent studies, vaccine stock-outs for routine childhood vaccinations have been steadily improving in many countries on account of improved vaccine need forecasting. Nevertheless, to ensure sustainable vaccine supply chains and to address the major challenges in ensuring access to vaccines, there is a need for long-term infrastructure investments that strengthen supply chain weaknesses and logistics. Efficient interventions that aim to decrease vaccine shortages are urgently needed, namely: [1] addressing delays in releasing national funds in a timely manner, [2] enhancing forecast precision, inventory management and data systems, [3] tackling tiresome national procurement procedures and delays, especially in countries that self-procure their vaccines [30]. Interestingly, PBF proved effective in increasing full immunization coverage, (as well as assisted deliveries and prenatal visit rates) when the funding was channeled through contracting NGOs in a PBC model [31, 32]. PBF is effective in improving quality of care and delivery of health services [13, 19, 20, 24, 33] and was found to increase a positive feedback loop on health worker motivation [34]. When designing incentives for PBF, healthcare workers’ lack of prioritization of financial incentives alone emphasizes the importance to identify the drivers of responsiveness to each incentive [26, 35]. In some situations, PBF arrangements may create ineffective parallel structures that might be unsustainable if they are not integrated into the national Public Financial Management (PFM) system. It is important to identify how PBF principles align with the budget and to ensure they are integrated into the national PFM system in order to avoid parallel systems that could otherwise be unsustainable [36]. We found PBF to have a mixed effect on antenatal and postnatal care visits, with a greater effect on rate of antenatal care visits [35], [25].

Demand side schemes that provide non-monetary incentives, commonly as food packages to encourage patients to pursue TB treatment and TB testing has been found effective in increasing rates of TB treatment completion. In CCTs where pregnant women were incentivized to give birth at a health facility, perinatal deaths as well as neonatal deaths were reduced, and rates of assisted deliveries were increased [29, 37]. In comparison to CCTs alone, voucher schemes incentivizing patients as well as healthcare workers proved to be more effective, especially in cases where women had to pay for transportation that they were not reimbursed for and in cases where women paid for services upon arrival [16]. CCTs create strong incentives to change behavior, however some perverse effects could arise such as in cases in Honduras and Brazil [38, 39] and that’s why measures of welfare during program design should be broad enough to record intended and unintended effects [40]. Factors that contributed to the impact of voucher schemes include a comprehensive voucher package that removed access barriers for patients [41]. Hybrid models combining the supply side -including a health system strengthening aspect-with a demand side incentive significantly increased rate of institutional deliveries and consequently reduced maternal mortality at acceptable costs [42],[22].

When looking at GAVI’s ISS program, funding continuation was conditional upon performance and healthcare quality with an extra USD 20 per child vaccinated, which kept the financing design simple and sent a message that the value of vaccinating a child is equal across contexts [43]. A key feature of this scheme was its flexibility as GAVI gave governments the power to choose how the funds will be disbursed [16]. It was found that low-income countries under stress, political instability and lower population growth rates were less likely to benefit from the ISS funding. The evaluation of outcomes was conducted through a one-time data quality audit until an independent study found discrepancies between household survey results and administrative reports, meaning that GAVI has been overpaying in some cases [43]. We also saw discrepancy between household surveys and healthcare facilities in other studies [20]. The concept of monitoring and evaluation is a repeated issue in DAH and this emphasizes that independent verification is needed whenever performance-based payments are introduced [43]. The ISS program has since been phased out and is now replaced with a new performance-based funding scheme under the Health Systems Strengthening (HSS) program.

SMI is considered to be a pioneer in the world of RBA in terms of the achievements it had in improving health system inputs [44]. This mode of disbursement gave recipients greater decision making power in where the funds should be dispersed [45]. RBA driven only by donors, or focused on one measurable result can lead to an increased risk of adverse incentives and non-systematic strategies [46]. Documentation for SMI stresses the involvement of each country in developing its own plan and coherence with domestic national health strategies, suggesting a great deal of recipient discretion. SMI was monitored using an independent third party [47], a monitoring procedure that probably contributed to its success [44, 48]. Regional mechanisms for sharing information by holding regular meetings to report about progress promotes healthy competition through peer pressure. Reinforcing that increasing visibility of results drives progress and pushes participating countries to meet deadlines. Knowledge sharing approaches help ensure efficiency and effectiveness of future DAH in global settings through the theory of change [43, 44].

### DAH and COVID-19

Despite the stark increase in development assistance towards COVID-19, the amount of health spending on COVID-19 was much higher in high income countries than in LMICs [49]. The COVID-19 pandemic and the global context in which it has spread has shown the significant inequalities at the national level within the health sector [50]. The report by the GPRBA suggests that COVID-19 will have significant implications for RBF programs in LMICs. The pandemic has highlighted the need to strengthen health systems and improve their resilience to future pandemics, while also ensuring that RBF programs are better equipped to respond to evolving health system needs. In order to address the new challenges posed by the pandemic, further outcome-based programs in LMICs should prioritize flexible and adaptive designs that can respond to crises like COVID-19 [51].Therefore, focusing support on healthcare quality, especially in this current climate with COVID-19’s huge global effects, a new DAH model that is focused on stronger and more resilient healthcare systems should be a priority [52].

### Factors affecting aid effectiveness

Multiple factors need to be kept into consideration that have implications on aid effectiveness such as [53]:

- Aid allocation, where aid is not based on need, but on donor interests. For example, donors at times place emphasis on specific targets such as communicable diseases and divert away from non-communicable diseases, thereby distorting national priorities.
- Aid fragmentation, which results in multiple projects and reporting systems, creating costs for recipients.
- Unpredictability of aid, which hinders long-term planning and can lead to aid being used for other purposes.
- Aid fungibility, which occurs when aid substitutes rather than supplements local spending.
- Mutual accountability is necessary for aid effectiveness but is often lacking.

Aid effectiveness can be improved by adapting best practices to local contexts, promoting local ownership, and improving coordination and accountability between donors and recipients. Furthermore, when implementing any type of a results-based funding approach tailoring financing packages to reflect the demographic and epidemiological profiles of the country, providing bonuses at the facility level and potentially using district health management teams to perform verification roles may help overcome such limitations[54]. In LMICs, attributing specific health outcomes to DAH can be challenging due to many reasons, some of which include gaps in models used to estimate outcomes, overestimated assumptions of financing effects and lacking reliable data [55]. It is important to note that donors are increasingly being called upon to adhere to aid effectiveness principles, which aim to strengthen country ownership and reduce parallel reporting and monitoring systems [56]. While these principles are important for ensuring DAH is utilized effectively, it is important to recognize that they may present challenges in terms of data collection and evaluation. Despite these challenges, adherence to aid effectiveness principles is crucial for promoting sustainable development and improving health outcomes in LMICs.

Implementing partners such as the WHO and UNICEF, to name a few, play a crucial role in the delivery of DAH, specifically in situations where there are concerns about the misuse of funds. However, working with implementing partners can also pose challenges, including increase in aid dependency, lack of sustainability and hindering of planned health system development of countries. Therefore, civil society organizations should also be included as implementing partners to ensure responsiveness to community needs and cultural appropriateness. In order to ensure the effective delivery of DAH, which ultimately benefits both donors and aid recipients, it is important to implement rigorous monitoring and evaluation systems [57].

### Negative Effects of Aid

Development assistance for health has resulted in significant progress towards improving health outcomes, but it has also contributed to the fragility of health systems and institutions in developing nations. The structure and motivations of development assistance have led to a reduction in funding for basic healthcare serviced in low-income countries, causing them to become overly reliant on external aid. This reliance can decrease their ownership of healthcare policy priorities and service delivery. The COVID-19 pandemic has demonstrated the dangers of depending too heavily on foreign financing and suppliers for essential healthcare needs. Instead of funding basic healthcare budgets, aid should be redirected towards financing regional and global public goods to enhance the accountability and ownership of healthcare expenditures and balance the power dynamic between the global south and north for the benefit of all[58].

### Study limitations

Our study has a number of limitations. Finding sufficient data to conduct a full comparative analysis was found challenging as the data allowed us to get a broader scope on what effects models have, but not enough to quantify the differences between financing models. Another limitation we faced is the low certainty evidence found in some of the studies. Due to the wide heterogeneity in study designs, research questions, interventions and outcomes reported, statistical pooling of all included results was not possible. However, we included an in-depth narrative synthesis which adds to the exiting evidence. One issue in the field of financing models is the lack of a standardized terminology, which can lead to confusion and misinterpretation in analyzing research findings. Furthermore, the lack of consensus on the language used to describe the varying financing models may lead to the misattribution of studies. This could undermine the accuracy and reliability of research in this field and make it difficult to draw solid conclusions or make informed decisions based on available evidence. Therefore, it is essential to establish a common vocabulary and a clear set of definitions for financing models to ensure proper categorization. We attempted to resolve this by following the working definitions provided at the beginning of the paper.

## Conclusions

Implementing a results-based approach is currently on the forefront of development assistance for health. However, the effectiveness of the different results-based approaches varies by context and health target. The available evidence suggests that aid provided for governments’ effectiveness is increased by good governance and accountability especially since results-based aid relationships are based on the relationship between donor development partners and recipient partner governments. Designing an RBF model that combines incentives to both the demand and supply sides demonstrated great effectiveness. Such voucher schemes are an effective solution to access barriers faced by incentivized patients such as transportation costs that could otherwise fall on them if incentivized solely by CCTs. Moreover, by addressing the supply side, these schemes can enhance healthcare quality and health capacity for incentivized patients. Different countries where similar models were implemented show differing results, further proving the great importance of understanding contextual factors surrounding each setting a financing model is applied in. In any case, when designing a financing model, focusing on health quality and strengthening of healthcare systems rather than specific indicators could be the most effective sustainable approach.

Last, it is imperative to emphasize the importance of including rigorous monitoring and evaluation plans, including if possible, an independent evaluator. Here, there is a great need for standardized indicators and methods to measure the effectiveness of DAH.

## Electronic supplementary material

Below is the link to the electronic supplementary material.

Supplementary Material 1

Supplementary Material 2

## Data Availability

All data generated or analysed during this study are included in this published article and its supplementary information files.

## Declarations

### Authors’ information

The authors include one female and one male and span multiple levels of seniority. The supervising author is a behavioral scientist with 18 + years of public health research experience in global settings. The other author is a Master of International Health Student in Berlin with clinical and research experience in the Middle East.

## Abbreviations

ODA: Official Development Aid
CRS: Creditor Reporter System
IDA: International Development Association
DAC: Development Assistance Committee
NGOs: Non- governmental Organizations
MDGs: Millenium Development Goals
OECD: Organisation for Economic Co-operation and Development
GAVI: Gavi, The Vaccine Alliance
SDG: Sustainable Development Goals
HIV/AIDS: Human Immunodeficiency Virus
COVID-19: Coronavirus
UN: United Nations
DAH: Development Assistance for Health
PEPFAR: President’s Emergency Plan for AIDS Relief
LMICs: Low-Middle income countries
IHME: Institute for Health Metrics and Evaluation
USAID: United States Agency for International Development
LICs: Low-income countries
TB: Tuberculosis
EC: European Commission
DFID: Department for International Development
RBA: Result-Based Aid
CGD: Center for Global Development
JSY: Janani Suraksha Yojana
CODA: Cash on Delivery Aid
IDOS: German Institute of Development and Sustainability
HF: Health Facility
RBF: Result-Based Financing
GPRBA: Global Partnership for Results-Based Approaches
ANC: Antenatal Care
PBF: Performance Based Financing
JBI: Joanna Briggs Institute
MCH: Maternal and child health
P4P: Pay for Performance
PRISMA: Preferred Reporting Items for Systematic Reviews and Meta-Analyses
DALYs: Disability- adjusted life year
CCT: Conditional Cash Transfer
ISS: Immunization Information Systems
PMTCT: Prevention of Mother to Child Transmission
PBC: Performance Based Contracting
SMI: Salud Mesoamerica
DOT: Directly Observed Therapy
ART: Antiretroviral therapy
ASHA: Accredited Social Health Activist
DPT3: Diphteria, Pertussis Tetanus Vaccine
HSS: Health System Strengthening
GDP: Gross domestic product
LICUS: Low-income countries under stress
PFM: Public Financial Management
