# Supplementary material for: Comparative effectiveness of financing models in development assistance for health and the role of results-based funding approaches: a scoping review
Source: Global Health. 2023 Jun 20;19:39. doi: 10.1186/s12992-023-00942-9 (PMC10283263; doi:10.1186/s12992-023-00942-9)
Supplement: Supplementary file 1 — Supplementary Material 1 [file 12992_2023_942_MOESM1_ESM.pdf]

Table 1: Extraction form of study design and interventions

	Author	Publication year	Study design	Study method	Timeframe	Type of financing	Country/Countries	Funding target	Mode of disbursement	Amount of disbursement	Funding source
1	Allegri *et al*	2019	quasi-experimental	Controlled interrupted time series	July 2012-March 2017	PBF&CCT	Malawi: Balaka, Dedza, Mchinji, Ntcheu	Maternal and neonatal health	Performance contracts to facilities and DHMTs Incentivize women for giving birth in health facility and spending 48 hours PP (combined supply side and demand side)		Ministry of Health in Malawi
2	Ashir *et al*	2013	quasi-experimental	two cell study design	November 2011 to April 2012	CCT (cash vouchers)	Nigeria: Yobe State	Maternal and child health	cash vouchers for receipt of 1 and 4 ANC visits; skilled delivery for women; and DPT3 and measles immunization for children.	N 500 (3 USD) for all targets besides skilled delivery N 1000 (6 USD)	Department of international development (UK) and the Norwegian government
3	Alfonso *et al*	2013	quasi-experimental	difference in differences impact, evidence based lives saved tool	June 2010-May 2011	PBF& vouchers	Uganda	Maternal mortality	voucher for roundtrip transportation and another voucher for delivery at HF, supply side support through HSS	transport voucher 1.8-4.5 USD<br>HF service voucher 2.8-5.75 USD<br>Referral to specialist voucher 27-59 USD	
4	Zeng *et al*	2018	quasi-experimental	pre and post household surveys and difference in differences impact	2012 to 2014	PBF	Democratic Republic of Congo	Maternal and child health services	incentives for services provided	Payment per unit range 0.4-20	World Bank

5	Lim *et al*	2010	quasi-experimental	collecting data from district level household surveys	2002-2004 and 2007-2009	CCT	India: high focus and non-high focus states	Maternal and neonatal health	incentivize women of low socioeconomic status to give birth in a health facility	generally, 600 Indian rupees in urban areas, 700 Indian rupees in rural areas	Indian government
6	Lagarde *et al*	2007	Systematic Review	Literature review	2004	CCT	Nicaragua	Improving access to health services and improve health outcomes	Disadvantaged households in low-income areas received a cash transfer provided they brought their children who were younger than 5 years to preventive health examinations and attended health education workshops	Mean $25, $18 per family, $9 per family with school-aged child, $20/y for supplies (around 20% of household consumption)	
						CCT	Honduras		two incentives: conditional on school attendance of children 6 to 12 years old and the other on undergoing monthly preventive health examinations for children, prenatal care attendance for pregnant women	Mean $17, $4 per family, $5 per child (almost 10% of household consumption)	
						CCT	Malawi		financial incentives to individuals who underwent HIV testing to increase collection of HIV virus test results	Mean $1.04, vouchers valued $0-$3 randomly assigned	
7	Diaconu *et al*	2021	Systematic review	Literature review	Updated in 2020	P4P (PBF)	Multiple	Health intervention delivery	performance based incentives	absolute magnitude range 0.5 USD and 10 USD per indicator	22 national governments<br>20 external agencies<br>4 external agencies in partnership with national entities
8	Steenland *et al*	2017	quasi-experimental	Difference in differences	2011-2013	PBF pilot	Burkina Faso: North (Titao), Center-North (Boulsa), and Center-West (Leo)	Maternal health services	incentives for the number of antenatal care visits, the proportion of antenatal care visits that occurred during the first trimester of pregnancy, the number of complicated and		commissioned by World Bank

									uncomplicated deliveries at the health facility, and the number of postnatal consultations occurring 42 days after pregnancy provided		
9	Rajkotia *et al*	2017	quasi-experimental	Difference in differences	18 months	PBF	Mozambique: Nampula (North) and Gaza (South)	provision of HIV, prevention of mother-to child HIV transmission (PMTCT), and maternal/child health (MCH)	based on the unit price of a service multiplied by the quantity of that service produced	as of 2014: 11 million USD in incentives	NGO partner funded by CDC
10	Gergen *et al*	2018	Qualitative study	Semi structured interviews and focus group discussions	November 2015-December 2015	PBF	Mozambique: Nampula (North) and Gaza (South)	provision of HIV, prevention of mother-to child HIV transmission (PMTCT), and maternal/child health (MCH)	based on the unit price of a service multiplied by the quantity of that service produced		NGO partner funded by CDC
11	Oxman *et al*	2009	Review	Critical appraisal of impact evaluations, key informant interviews		PBF&CCT	India	Maternal and neonatal health	PBF: ASHA receive performance-based compensation for promoting a variety of primary healthcare services in general<br>CCT: incentives for mothers to give birth at an institution	200 to 1400 rupees ($4.94 to $34.58) for institutional delivery for mothers and ASHA, adjusted for rural and urban areas.	Indian government
						Demand side incentives	Tajikistan	Tuberculosis control	Food support is provided to DOTS patients who adhere to treatment and their families who are considered vulnerable, program then expanded to include almost all patients		USAID with Project home and WFP
						RBA	52 countries' national governments	Immunization coverage	The imputed cost of immunizing an additional child was approximately $23 at	145 million USD in June 2006, estimated 15 % increase	GAVI

									the lowest coverage rates. Once coverage rates were above 60 to 70%, the cost per child immunized increased exponentially.		
12	Turcotte-Tremblay *et al*	2016	Systematic Review	Literature review	January 2012 and June 2014	PBF	Haiti	Growth of services	International support (training and monitoring) and incentives on services provided		USAID
							Rwanda	Maternity and child care	Incentives to providers	Administrative costs estimated at US$0.3 per person in total	NGOs then implemented by National programs
13	Van de Poel *et al*	2015	quasi-experimental	data from the Cambodian Demographic and Health Survey (CDHS), difference in differences linear probability model	2000, 2005, 2010	PBF, PBC	Cambodia	Maternal and child health services	Fixed price contract with progress payments contingent on performance against service targets, then performance contract with MoH specifying targets but w/o incentive payments then payment per unit of service delivered then facility enters performance contract with SOA with incentive payments		NGO<br>MoH with NGO advisors<br>GAVI<br>SOA with MoH
14	Eichler *et al*	2009	Case study	independent survey research firm, exit interviews in service delivery institutions, houehold interviews and sample records, family planning registers review, measuring waiting times	three phases: Pilot (1999), second and third phases (2000-2004, 2005-2007)	PBC	Haiti	maternal and child health, reproductive health, and family planning services	NGOs are paid partially based on achieving defined performance targets related to attainment of health output targets and strengthening of institutional capacity		USAID

15	Eichler, Levine and the performance incentive working group	2009	Case study			PBF Pilot	Rwanda: Cyangugu	quality of curative, maternal and child health, and HIV/AIDS services	payments were made directly to the facility, with health committees or management deciding how to use funds; on average, roughly 40 percent was given as staff bonus payments and 60 percent was reinvested at the facility.		Healthnet<br>Cordaid<br>Belgian Technical Cooperation (BTC)
						PBF Pilot	Rwanda: Butare		health centers had to inform the steering committee in advance of the pay scale for bonus payments, the money was given to the health committee, which then paid the staff.		
						PBF	Rwanda: Kigali-Ngali, Kabgayi, and Kigali Ville		facilities received payments and distributed them among personnel according to previously agreed criteria that captured the relative contributions of staff		

						CCT&PBF	Nicaragua	Basic health and nutrition services and education	cash transfers to households for health, given to the mother when possible, conditional on attending health education workshops and taking children under 5 for mandated health care appointments<br>healthcare providers were paid to deliver the services covered by the program free of charge	CCT: US$224 per year, paid every 2 months Supply side: per capita payment of US$130 per year per household<br><br>phase 2 the amount of the demand side transfer was reduced by 30% , the supply side performance based payment remained the same,	RPS with IDB
						Demand side incentives	Tajikistan	Tuberculosis case detection and treatment adherence	Food packages are provided to vulnerable patients and their families on a bimonthly basis conditional on adherence to treatments.<br>Providers maintain and review treatment cards to determine adherence. Food packages contain wheat flour, vegetable oil, pulses, and salt	The package value is approximately $172, which, for the average-size Tajik family, is equal to about $29 per person for the six-month course of treatment	collaborative work of NGOs with financing by the USAID

						PBF	Bangladesh	Tuberculosis case detection and treatment adherence	Patients pay a deposit upon initiation of treatment and receive 37.5% back at completion of therapy; community supervisor receives remainder of deposit. (Patients assume financial risk-scheme tied to performance)		NTP
16	Eichler, Discussion paper for the first meeting of the Working Group on Performance-Based Incentives Center for Global Development					Demand side incentives	Cambodia	Tuberculosis case detection and treatment adherence	Food is provided to in-patients in hospitals and food packages to outpatients who attend clinic for treatment		Food from World Food Programme (WFP). NTP
						CCT	India: Cochin		Monetary support is provided to patients to enable travel, to purchase food and as an incentive to motivate behavior.		
						Demand side incentives	Sudan		Patients are provided with food packages and transport to DOTS centers.		Food from World Food Programme (WFP). NTP
						Demand side incentives	Yemen		Food is provided on a monthly basis to patients who attend the clinic for treatment.		NTP

17	Beane *et al*	2013	Systematic Review	Literature review and informant interviews		RBF (PBF, PBC,CCT)	Rwanda, Democratic Republic of Congo, Egypt, Burundi, Tanzania, Cambodia, Liberia, Afghanistan, Southern Sudan, Uganda,				
18	Eldridge, Palmer	2008	Systematic Review	Literature review		PBC	Cambodia				
						PBC	Haiti				
19	Mokdad *et al.*	2018	Quasi experimental	Health facility surveys involving an interview questionnaire, an observation checklist, and medical record reviews	18 to 24 months follow-up	RBA	Mesoamerica	maternal and child health	Participating countries receive 50% of the cost of program intervention from funders and contribute the remaining 50% themselves. At the end of each operation, pre-defined performance indicators are measured independently, and if 80% of these indicators are met, the country is awarded half of its contribution share back.	23 Million USD for first operation	IDB
