# Supplementary material for: Comparative effectiveness of financing models in development assistance for health and the role of results-based funding approaches: a scoping review
Source: Global Health. 2023 Jun 20;19:39. doi: 10.1186/s12992-023-00942-9 (PMC10283263; doi:10.1186/s12992-023-00942-9)
Supplement: Supplementary file 2 — Supplementary Material 2 [file 12992_2023_942_MOESM2_ESM.pdf]

Additional file 2: Data extraction form of the outcomes aggregated by indicator

Table 1: Percentage increase of rates of attending institutional/assisted deliveries

Country	Financing model(s)	Outcome(s)
Nigeria	CCT	4.5%
Democratic Republic of Congo	PBF	42%
Burkina Faso	PBF	9.2%
Rwanda	PBF	23%
Rwanda: Cyangugu, Butare	PBF	10.9%
Rwanda: Kigali-Ngali, Kabgayi, and Kigali Ville	PBF	8.5%
Cambodia	PBF&PBC	Estimated 25% increase
India	PBF&CCT (combined supply and demand side)	32.6 % increase<br>One year after establishment of program: 36% increase
Haiti	PBC	2%

Table 2: Care visits (preventive/antenatal/prenatal/postnatal)

Country	Financing model(s)	Outcome(s)
Nigeria	CCT	The intervention area consistently registered a statistically significant higher proportion of women with 4 ANC visits than their counterparts
Democratic Republic of Congo	PBF	Relative increases in curative care by 83%
Honduras	CCT	• Percentage of individuals receiving pre-natal care: 19 pp increase<br>• Routine pediatric examinations increase: 20 pp increase

		• Growth monitoring by visits for children: 16 pp increase<br>• No effect on percentage of women who received a 10-day follow-up visit after delivery
Burkina Faso	PBF	• Relative increase of 27.7 percent for ANC<br>• Relative increase of 118.7 percent for postnatal care
Mozambique: Nampula (North) and Gaza (South)	PBF	Women completing 4 ANC visits:<br>North: 153% increase<br>South: 82.4% increase
Rwanda	PBF	• 56 % increase in preventive care visits by children aged 23 months or younger<br>• 132 % increase in preventative care visits by children between 24 and 59 months<br>• No improvement in the number of women receiving any prenatal care, the number of women completing four or more prenatal visits

Table 3:Immunization rates

Country	Financing model(s)	Outcome(s)
Nigeria	CCT	No significant effect on measles immunization
Nicaragua	CCT	Failed to demonstrate improved vaccination coverage
Honduras	CCT	Mean increase 6.9 pp in coverage of first dose of diphtheria, tetanus toxoids and pertussis
Nicaragua	CCT&PBF	Percentage of children 12-23 months with updated vaccinations

		Baseline: control: 41.5%<br>intervention: 38.9%<br>Follow up: control: 69.4%<br>intervention: 71.4%
Haiti	PBC	Full immunization coverage<br>year 2000: 34 %<br>year 2005: 41.3 %
Haiti	PBC	Increased immunization coverage from a baseline of 42% to 74%
Democratic Republic of Congo	PBF	Did not improve full immunization among children and anti-tetanus vaccination (VAT2þ) among pregnant women
Rwanda	PBF	No improvement in the number of children receiving full immunization schedules
Rwanda: Cyangugu, Butare	PBF	Measles immunization:<br>Before (2001): 70.7 %<br>After (2004): 81.5 %
Rwanda: Kigali-Ngali, Kabgayi, and Kigali Ville	PBF	Immunization:<br>Before (2004): 80.0 %<br>After (2005): 83.6%
52 countries' national governments	RBA	A relationship was found between ISS funding and increased immunization coverage.

Table 4: Mortality rates

Country	Financing model(s)	Outcome(s)
Malawi: Balaka, Dedza, Mchinji, Ntcheu	PBF&CCT	Reduced facility based maternal mortality by 4.8 deaths/100 000 facility - based deliveries
Uganda	PBF& vouchers	This 9.4% bump in institutional delivery implies 20 deaths averted, which is equivalent to 1356 disability- adjusted-life years (DALYs) averted
India	CCT	Matching:<br>Reduction: 3.7 perinatal deaths/1000 pregnancies

		2.3 neonatal deaths/1000 livebirths<br>With-versus without comparison: reduction of: 4.1 perinatal deaths/1000 pregnancies 2.4 neonatal deaths/1000 livebirths
Tajikistan	Demand side incentives (food packages)	2.9 percent of patients in the food support group died versus 12.5 percent in the comparison group

Table 5: Case detection and testing rates

Country	Financing model(s)	Outcome(s)
Democratic Republic of Congo	PBF	Increase HIV/AIDS testing among pregnant women (147%)
Malawi	CCT	Individuals collecting HIV test results mean percentage increase by 27%, positive linear effect with level of incentive
Bangladesh	PBF	Tuberculosis case detection rate= 50%.<br>Reported results are partially attributed to incentive scheme.
India: Cochin		Increased Tuberculosis case detection rates

Table 6: Child health indicators

Country	Financing model(s)	Outcome(s)
Nicaragua	CCT	Reduction in magnitude of stunting (net mean improvement of the height-for-age z score by 0.17)<br>After 2 years: net impact of 6 pp in proportion of underweight children aged 0 to 5 years<br>No impact on anemia prevalence among infants
Nicaragua	CCT&PBF	Percentage of stunted children under 5:

		Baseline: control: 39. 5% intervention: 39. 8% Follow up (2002): control: 41.7% intervention: 36.5 %

Quality of care and service delivery

Country	Financing model(s)	Outcome(s)
Democratic Republic of Congo	PBF	Increase in patient referrals (472%), vitamin A distribution (155%)
Haiti	PBF	Incentives alone were associated with a 39 % increase in health serv ices. Support alone was associated with a 35 %increase in health services. Support and incentives were associated with an 87 % increase compared with health facilities that did not receive either.
Haiti	PBC	Increased oral re-hydration salts usage

Table 7: Patient motivation

Country	Financing model(s)	Outcome(s)
Uganda	PBF& vouchers	Demand for births at HFs enrolled in the voucher scheme increased by 52.3 percentage points. Out of this value, conservative estimates indicate that at least 9.4 percentage points are new HF users.
Nicaragua	CCT	Mean increase in proportion of infants (aged 0-3 years) taken to health centers in the past 6 months<br>1 year: 19 pp<br>2 years :11 pp
Honduras	CCT	Significantly increased use of health services by 23% for infants younger than 1

		year and 42% for preschool children aged 1 to 5 years
Rwanda: Cyangugu, Butare	PBF	Family planning acceptors:<br>Before (2001):1. 1%<br>After (2004): 3.9 %
Rwanda: Kigali-Ngali, Kabgayi, and Kigali Ville	PBF	Family planning<br>Before: (2004):10.6 %<br>After: (2005):15. 7%

Table 8: Health worker motivation/satisfaction

Country	Financing model(s)	Outcome(s)
Mozambique	PBF	Internal drivers: enhanced self-efficacy driven by goal orientation, healthy competition among colleagues, and job satisfaction.<br>External drivers included an organized work environment, enhanced access to equipment and supplies, financial incentives, teamwork, and regular consultations with verifiers
India	PBF	43% of ASHA were satisfied and 36% were somewhat satisfied with the remuneration received
